# Centres of rotation and osteological constraints on caudal ranges of motion in the sauropod dinosaur *Giraffatitan brancai*

**DOI:** 10.1098/rsos.250851

**Published:** 2025-08-13

**Authors:** Verónica Díez Díaz, Pasha A. van Bijlert, William Irvin Sellers, Mathew J. Wedel, Daniela Schwarz

**Affiliations:** ^1^ FB1, Museum für Naturkunde - Leibniz-Institut für Evolutions- und Biodiversitätsforschung, Berlin, Germany; ^2^ Department of Earth Sciences, Faculty of Geosciences, Utrecht University, Utrecht, The Netherlands; ^3^ Naturalis Biodiversity Center, Leiden, The Netherlands; ^4^ School of Earth and Environmental Sciences, University of Manchester, Manchester, UK; ^5^ Western University of Health Sciences, Pomona, CA, USA

**Keywords:** *Giraffatitan brancai*, Sauropoda, tail, ranges of motion, centres of rotation, osteological limits

## Abstract

Archosaur tails are important appendages, not only for their biomechanical function but also for being behavioural tools that help the animal communicate and interact with its environment. Until recently, tails have been neglected in biomechanical analyses and were considered as a stiff (sometimes independent) unit; however, the tail’s role in movement is now increasingly being appreciated. In this work, we present detailed analyses of the ranges of motion of the amphicoelous anterior caudal series MB.R.2921 from the Late Jurassic sauropod *Giraffatitan brancai* from Tanzania. We discuss possible positions of the centres of rotation, potential osteological constraints and how they affect the mobility of the caudal series. Our results highlight the importance of considering haemal arches as functional units and osteological constraints in ventroflexion of the tail. Thorough range of motion analyses of the axial skeleton have the potential to yield novel insights into the functional morphology and behaviour of extinct animals.

## 1. Introduction

Although tails precede the evolution of paired appendages in vertebrates by approximately 200 million years [1,2], we are just starting to understand their function, development and evolution. For example, Schwaner *et al.* [2] highlight the importance of the tail regarding the physiological costs and benefits of using caudal and paired appendages together. However, because of the inherent complexity of segmented anatomical structures, the axial skeleton—and in particular the tail—is often ignored in musculoskeletal reconstructions and biomechanical analyses [2,3] or simplified as a stiff element (e.g. [4–6]).

This situation is different for the neck, as its functional relevance has been recognized in numerous studies, especially in the context of cervical elongation (e.g. giraffes, sauropod dinosaurs and Mesozoic reptiles [7–20]) or those with highly specialized anatomical features and motions (e.g. sloths and owls [21–23]).

Although the importance of the tail for understanding the locomotion and behaviour of tetrapods has been previously recognized (e.g. [4,24]), relatively few biomechanical analyses have focused on the tail, presumably due to its anatomical complexity [2].

Tails of extant tetrapods can have a wide range of functions (see [25] for an comprehensive list), being used, for example, as prehensile tools or to maintain balance in an arboreal [26,27] or terrestrial ([28] and references therein) lifestyle, as a propulsion organ for swimming ([29–31] and references therein), to improve terrestrial and aerial manoeuvrability, re-orientation and gliding [28,32–36], to divert a predator’s attention to a dispensable body part (i.e. the tail itself) [37] and as communication tools in social interactions (e.g. [38–41]). For terrestrial locomotion, the tail can also provide challenges, as Willey *et al*.’s [42] experiments demonstrated: although it provides stability, tail drag introduces costs in terms of propulsion and energy efficiency at slow speeds. Indeed, this might be the reason why galloping crocodiles are reported to extend their tail, presumably to improve their running performance [43,44].

Reconstructing potential biological roles of the tail for extinct tetrapods requires a thorough biomechanical analysis of the ranges and constraints in tail mobility. Recent studies have highlighted the importance of tail biomechanics for reconstructing dinosaur locomotion. For example, passive-dynamic effects in the tail of *Tyrannosaurus rex* may provide a good indicator for the energetically optimal walking speed of bipedal dinosaurs [45]. The unique tail anatomy of the theropod clade Alvarezsauria possibly indicates an exceptional capacity to change rotational inertia, enabling sharp turns [33], and muscle-driven simulations of the bipedal theropod *Coelophysis* suggest that lateral flexion would have reduced muscle effort during locomotion [46]. Beyond locomotion, Arbour & Snively [47] examined the distribution of stress in simulated ankylosaurid tail club impacts, while Lategano *et al.* [48] investigated stress resistance of a stegosaurian tail using multi-body dynamics analysis. Conti *et al.* [49] evaluated whether the tails of diplodocid sauropods could act as a defensive weapon by reaching the speed of sound. Recently, several works have calculated the ranges of motion (RoMs) of some titanosaurian caudal series [50–54], informing postural and behavioural hypotheses.

Recent studies have explored axial mobility in tetrapods from both morphological and computational perspectives. For example, several works [7,8,14,15] combine anatomical reconstructions with geometric modelling [55] and data from living forms [56] to infer patterns of mobility. Research on the caudal region of sauropods [51–54] explores the importance of tail biomechanics in locomotion, supported by digital reconstructions [50] and comparative studies. Finally, computational techniques such as finite element analyses (FEA) and automated analyses [17,23,57] highlight the value of digital tools for understanding the evolution of axial mobility.

In this work, we analyse the RoMs of the caudal series MB.R.2921 of *Giraffatitan brancai*, a titanosauriform sauropod from the Late Jurassic of Tanzania [58]. We test how the location of the centre of rotation (CoR) between adjacent vertebrae impacts the RoMs of sauropod dinosaurs with non-concavo–convex joints, as already confirmed in humans, cats and theropod dinosaurs (e.g. [45,57,59] and contra [57]; see below). In addition, we highlight the most important osteological constraints on tail movement, including the haemal arches. All this data will provide a more objective framework for exploring the biological roles of the tails of dinosaurs and other extinct terrestrial tetrapods.

*Institutional abbreviations*: MB.R., Collection of Fossil Reptiles, Museum für Naturkunde Berlin, Berlin (Germany); MWC, Museum of Western Colorado, Fruita, Colorado (USA).

## 2. Material

**Figure 1.**
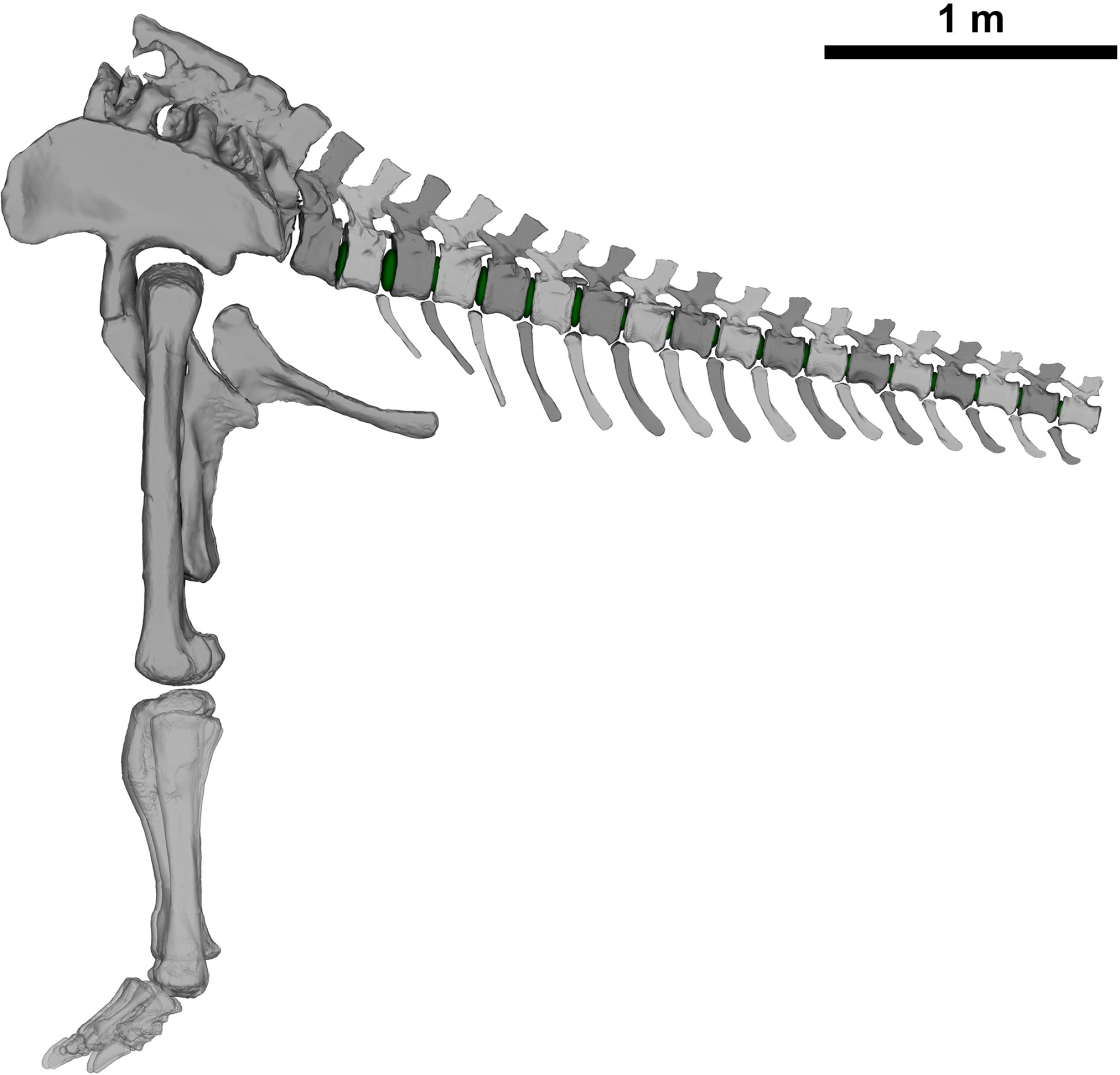
ONP of the MB.R.2921 caudal series of *G. brancai*, with (green) intervertebral discs between vertebrae (modified from Díez Díaz *et al.* [3]).

The caudal series MB.R.2921 of *G. brancai* was used to analyse its RoMs (figure 1; electronic supplementary material, table S1). This series consists of the first 18 caudal vertebrae (MB.R.2921.1-18) and 14 haemal arches (MB.R.2921.19-32), found in articulated sequence following posterior to the last sacral [58].

### 2.1. MB.R.2921 caudal vertebrae morphofunctional units

Sauropod caudal vertebrae generally present a gradual morphological change along the tail, varying in the length and proportions of the centra, neural arch size and orientation of the neural spines, elongation of the zygapophyses, and presence and morphology of the transverse processes. These anatomical features are of great importance for assessing osteological correlates of muscle attachments [3], as well as for evaluating possible motion limits in the articulation between vertebrae [9,15,50,60]. Three different morphofunctional units or regions (i.e. modules) have been identified along the necks of birds (see [61] and references and discussion therein). It is recognized across sauropsids that tail functioning changes from proximal to distal. Luger *et al.* [62] observed regionalization in the tail of chameleons. Studies show that the crocodile tail comprises two different functional sections that help the animal change directions and support propulsion in aquatic environments [63]. Therefore, differentiating sections within the caudal series helps to assess potential serial changes in the motion capabilities of the tail.

Russell [64] defined the term ‘transition point’ as the change that occurs between the anterior and posterior segments of the tail of ornithomimosaurian theropods, where the last caudal vertebrae possessing transverse processes meet the first with clearly elongated zygapophyses. This transition point has been recognized in many other theropod groups (e.g. [33,45,65,66] and references therein) based on loss of transverse processes, extreme reduction of the neural spines, elongation of the caudal centra and haemal arches with an inverted T-shape [66–68]. Moreover, the most distal attachment of *M. caudofemoralis longus* is hypothesized to occur close to this transition point [69,70]. The placement of the transition point in the caudal series differs across clades; this, together with the musculoskeletal changes that occur in the tail beyond this point, most likely reflect significant biomechanical and functional modifications (e.g. [33,71]).

**Figure 2.**
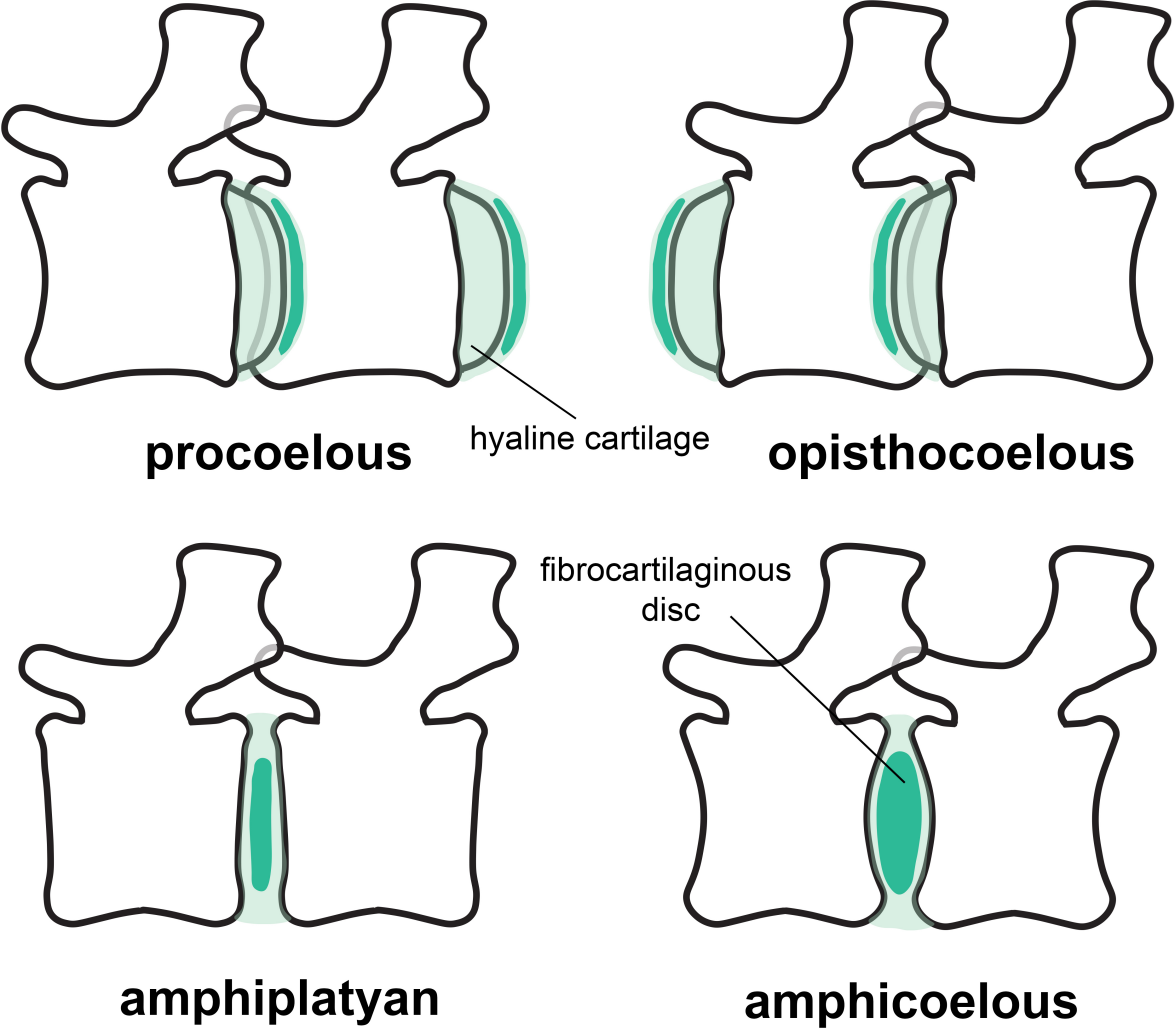
Types of articulations in sauropod caudal vertebrae and intervertebral cartilage. Concavo-convex articulations (procoelous at the left and opisthocoelous at the right) are depicted at the upper half of the figure, while amphyplatyan (left) and amphicoelous (right) are depicted at the lower half. Note that procoelous, opisthocoelous and amphiplatyan vertebrae can present synovial joints or fibrocartilaginous discs, while amphicoelous vertebrae always require the presence of thick fibrocartilaginous discs because of the larger intervertebral space.

Three caudal series are known for the sauropod *G. brancai*: MB.R.5000 (50 caudal vertebrae, from the second to the fifty-first), MB.R.3736 (29 caudal vertebrae, from the second to the twenty-fourth, and from the twenty-sixth to the thirty-second) and MB.R.2921 (18 caudal vertebrae, from the first to the eighteenth; see [3] for more detailed information on these caudal series). A probable transition point can be observed in MB.R.3736, between the 17th (MB.R.3637.17) and 18th (MB.R.3637.18) caudal vertebra [3, fig. 2D]; however, this caudal series is poorly preserved, so the presence and the accurate placement of this point are ambiguous. In this work, we rely on the caudal series MB.R.2921 described by Díez Díaz *et al.* [72], and Mannion *et al.* [73] for differentiating those regions, as they are clearly preserved in this series (a more detailed description of the anatomical features of the caudal vertebrae of the MB.R.2921 series is provided by Díez Díaz *et al.* [3]):

(1) First postsacral (or most anterior) caudal vertebra (Cd1): first caudal vertebra, with a triangular and laterally projecting transverse process; tall neural spine with laminae; without haemal arch articular facets.
(2) Anterior caudal vertebrae (Cd2–Cd18): with transverse processes and articular facets for the haemal arches. Neural spines become subsequently shorter, and the width of the transverse processes decreases in the distal direction within the series. Prezygapophyses become larger and less robust from Cd2 onwards.

Because the anterior sections of these tails are so well preserved, this analysis focuses on the region between Cd1 and 18, which represents the region before the transition point. This region serves as the attachment area for *Mm. caudofemorales* and as the functional base for the remainder of the tail. It is therefore important for hindlimb propulsion and for determining the overall mobility and stabilization of the tail. We reconstruct the RoMs of the anterior tail region of *Giraffatitan* and compare them with those of other sauropods, including the titanosaurs *Lirainosaurus* [50]*, Arrudatitan* [51], *Trigonosaurus* [52], *Adamantisaurus* and *Baurutitan* [53].

## 3. Reconstruction of intervertebral cartilage

### 3.1. Anatomical background on types of articulation

Intervertebral cartilage is a soft tissue that is usually not preserved in fossil specimens. To reconstruct the intervertebral cartilage in the caudal vertebrae of *G. brancai*, we used the anatomical data known for extant birds and crocodilians as the closest relatives of sauropod dinosaurs, as well as data from other tetrapods with similar osteology of the intervertebral articulations. Intervertebral articulations in tetrapods can be developed either as synovial joints [63,74] or as symphyses. Synovial joints (e.g. zygapophyseal articulations and articulations between limb bones) are mobile joints with a synovial membrane and synovial fluid that allow mobility in all three degrees of freedom. However, joint mobility is usually constrained by the architecture of the joint, its geometry and the mechanical properties of the soft and hard tissues, so that often only one or two degrees of freedom are reached. In contrast, symphyses (e.g. in the intramandibular joint and the intervertebral discs [IVDs]) include a fibrocartilaginous *annulus fibrosus* connected to the bone via the enclosing articular capsule and Sharpey’s fibres [74–76], and generally allow smaller RoMs than synovial joints.

A closer look into the intervertebral articulation of extant birds and crocodilians reveals that there is great plasticity both in the joint morphology and soft-tissue anatomy (i.e. the development of synovial joints and IVDs; figure 2). Birds generally possess synovial intervertebral joints in the cervical and anterior thoracic vertebral column (the joints between the middle thoracic vertebrae are often obliterated by fusion of adjacent vertebrae to form the notarium [77]). The articular surfaces of the cervical and anterior dorsal vertebrae in birds are covered with hyaline cartilage. The articular surfaces of the centra may articulate directly with one another, or the joint space may be divided by a thin meniscus of fibrocartilage [77,78]. Examples of the latter are present in *Struthio camelus* [13], *Cygnus atratus* [79] and *Aptenodytes patagonica* [80]. These fibrocartilaginous menisci have sometimes been referred to as IVDs (e.g. [80,81]), but this is a misnomer, confusing a meniscus within a synovial intervertebral joint with a non-synovial fibrocartilaginous symphysis [13,74]. Interestingly, and relevant to this study, the free caudal vertebrae of birds are described as having symphyseal joints [77,82], but unfortunately, it is not specified if the fibrocartilaginous discs in the tails of birds possess a *nucleus pulposus*, which forms the core of the IVD in mammals.

**Figure 3.**
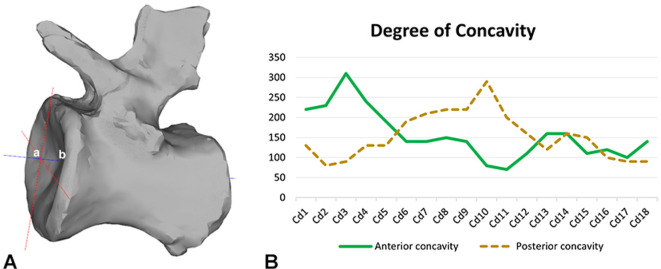
(A) graphical explanation for calculating the degree of concavity; the distance between a and b (in blue) defines this depth. (B) Concavity ratios of the anterior and posterior surfaces of the caudal vertebrae of the MB.R.2921 caudal series of *G. brancai*.

In contrast, crocodilians, which possess procoelous vertebrae through most of the vertebral column (transitioning into platycoelous articulations only in the terminal half of the caudal vertebral column), are described and figured to possess synovial joints [63,74] (contra [13]).

Synovial joints have been predicted for spherical, concavo–convex (such as the procoelous and opisthocoelous vertebrae in sauropods) intervertebral articulations with a short distance between condyle and cotyle, because there is almost no space for IVDs [63,74]. They also occur in the heterocoelous, saddle-shaped intervertebral articulations (*articulationes sellares* [77,78]) in the presacral vertebrae of most birds. However, a few birds (penguins, some charadriforms and some parrots; see [77] and references therein) have indeed opisthocoelous and therefore concavo–convex intervertebral articulations in the thoracic vertebrae. A bird-like type of interarticular connection with an IVD lacking a *nucleus pulposus* has been suggested for derived non-avian theropod dinosaurs (dromaeosaurids [74]).

Mechanically, concavo–convex articulations are highly stable and resistant to dislocation [63,83,84], so that flexible cartilage tissue between articulations could help facilitate the motion between vertebrae. In contrast, in amphicoelous and platycoelous vertebral articulations, such as in mammals, this intrinsic stability is reduced, and it is assumed that the IVD proper (consisting of an *annulus fibrosus* enclosing a *nucleus pulposus*) is necessary to withstand the stress applied to the vertebral bodies during locomotion while maintaining mobility [74,85]. In MB.R.2921, the caudal series of *G. brancai*, several degrees of amphicoely are prevalent (figure 2; electronic supplementary material, table S2; see §5). Deeper anterior surfaces are generally found in the anterior caudal vertebrae, while deeper posterior surfaces are found between Cd6 and Cd12 (see below). The volume enclosed by the amphicoelous articular surfaces suggests that, instead of synovial joints, symphyses in the form of IVDs were present in MB.R.2921. The presence of an IVD would, besides facilitating motion between vertebrae, also increase the stability and resistance of the articulation between two vertebrae. The presence of fibrocartilaginous, ellipsoid IVDs was previously hypothesized by Wedel *et al.* [86] for the deeply amphicoelous caudal vertebrae of the *Haplocanthosaurus* caudal series MWC 8028 [87], and it was suggested that they could also be present in more non-avian dinosaurs, such as in the caudal series MB.R.2921 of *Giraffatitan*. This reconstruction is also supported by histological evidence for an IVD in fossil reptiles, including non-avian dinosaurs, ichthyosaurs, plesiosaurs and marine crocodiliforms (see [74] for a more complete list and discussion), as well as by the presence of fibrocartilaginous IVDs in the avian caudal vertebrae [77,82]. As extant birds demonstrate, both synovial and symphyseal intervertebral joints can be present in different regions of the vertebral column in a single individual [86]. Similarly, *Giraffatitan* possesses opisthocoelous cervical and thoracic vertebrae, but amphicoelous vertebrae in the tail, potentially implying synovial intervertebral joints in the presacral column but fibrocartilaginous IVDs in the caudal series.

### 3.2. Joint articulations in the *Giraffatitan* tail model

Ellipsoid elements were created in the software Rhinoceros 5.0 (McNeel Associates; see below) in each articulation of the MB.R.2921 caudal series, following the morphology of both anterior and posterior surfaces of the centra (figure 3; see also [88]). The thicknesses of the modelled ellipsoid elements varied continuously throughout the tail, following the centrum-to-centrum distances in the osteological neutral pose (ONP); however, it is not possible to reconstruct the exact expansion of the IVDs or their composition (e.g. presence of an *annulus*/*nucleus* structure). We finally assume that the IVD would have articulated with hyaline cartilage, which formed the endplates of the vertebrae, as in extant taxa with IVDs (e.g. [89,90]). The centroids of these ellipsoid elements formed our baseline CoR (see below). In this study, we focused on the osteological limits on RoMs, because osteological limits form a more objective criterion than any attempt to account for the effect of IVD deformation on RoM. We assumed the cartilage would deform as much as required until an osteological limit was reached. The presence of hyaline cartilage is suggested for the articulations of zygapophyses and haemal arches, which, by comparison with extant tetrapods like crocodilians, birds and mammals, are reconstructed to be synovial joints [75,82,91].

**Figure 4.**
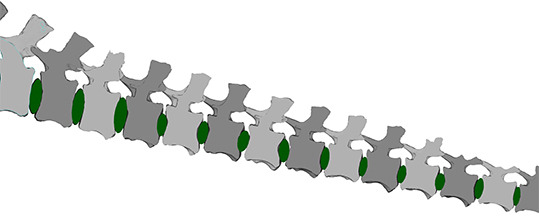
Transversal cross-section of the MB.R.2921 caudal series of *G. brancai*. Details on the modelled (green) ellipsoids as IVDs between the amphicoelous articular surfaces of the centra. As indicated in the text, these IVDs are considered fibrocartilaginous and embedded within hyaline cartilage.

## 4. Methods

The caudal series used in this work is the same previously published by Díez Díaz *et al.* [3]. Only some highlights on the digitization and three-dimensional (3D) assemblage workflows and protocols used for creating the digital caudal series will be detailed here; the reader is referred to the previous paper for a more in-depth description of the methods.

### 4.1. Digitization

The fossils were digitized by photogrammetry, following the protocol of Mallison & Wings [92]. Some haemal arches (MB.R.2921.19-20, 24, 29-30, 31) were digitally restored in zBrush 4R7 (Pixologic) because of their preservation, either by mirroring the preserved ramus or by scaling and superimposing the distal part of the blade of the adjacent haemal arch, and two missing haemal arches (the first and the twelfth) were entirely created digitally (see [3] for a more detailed information on these reconstructions). The 3D models were exported as OBJ files (between approx. 1500 and 50 000 polygons).

### 4.2. Assembling the caudal series

The 3D models were imported into Rhinoceros 5.0 and articulated in the ONP (after [19,20,60,93,94]) following the protocol described by Mallison [60,93]: individual vertebrae were articulated in pairs to minimize the effects of preconceived ideas (e.g. overall downward position of the tail, as in the former reconstruction of the mounted skeleton of *Giraffatitan*). Díez Díaz *et al.* [3] used a less taphonomy-influenced reconstruction of this caudal series (with small intersections between some caudal vertebrae), so a more realistic 3D musculoskeletal reconstruction could be created. In this work, we will use the 3D model created in [3], in which the prezygapophyses and postzygapophyses are used as proxies for assessing the correct articulations between vertebrae, assuming maximal zygapophyseal overlap (but see below) and—as far as possible—sub-parallel intervertebral faces. Haemal arches were positioned into the existing articulated caudal vertebral series by matching their anterior and posterior articular facets with the orientation of the ventral articulation surfaces of each vertebral centrum, thereby bridging the gap between a pair of subsequently following vertebral centra ventrally. A short space was retained between the proximal articulation surface of the haemal arch and the haemal arch articulation surface of the vertebra, representing the volume of the articular cartilage between the haemal arch and the vertebral centrum.

The caudal vertebrae of *Giraffatitan* possess postzygapophyses with a doubled articular surface (figure 4). A small oval-to-subcircular (depending on the development and position of the postzygapophysis) well-defined articular surface is located at the distal end of the postzygapophyses. This surface is where the distal area of the prezygapophysis fully articulates with its previous counterpart. The small surface is set within a larger oval one, and together they constitute the postzygapophyseal articular surface of the caudal vertebrae. This large double articulation area assists the prezygapophysis gliding into dorsiflexion, and its area is one of the osteological limits that define the motion (see below). This area is positively correlated with the length of the following prezygapophysis, generally allowing a greater dorsal RoM in the middle to posterior caudal vertebrae (figure 4; but see also the results of this work below, regarding CoRs). To our knowledge, this double surface has not been described before in any other vertebrate. We postulate that this feature is of biomechanical relevance and promotes dorsiflexion of the caudal vertebral column as described above. We suspect that the double surface of postzygapophyses is present in other sauropods and has simply escaped detection before now; this hypothesis can and should be tested in future descriptive and analytical work.

**Figure 5.**
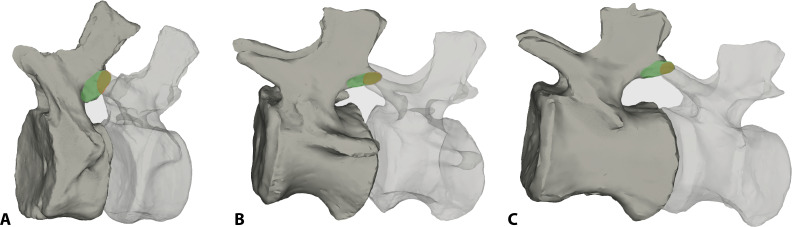
Doubled articular surface present in the postzygapophyses of the MB.R.2921 caudal series of *G. brancai*. The smaller oval-to-subcircular distal area is coloured in yellow, while the larger one is depicted in green; these areas have been here presented for the zygapophyseal articulations between (A) the caudal vertebrae 2 and 3, (B) the caudal vertebrae 10 and 11, and (C) the caudal vertebrae 16 and 17, showing this change on the outlines. The specimens are not to scale.

The taphonomic distortion mainly comprises slight plastic deformation in several postzygapophyses (posterior extension of left elements) and a lateral translation of the prezygapophyses of Cd17 and Cd18, leading to a slight asymmetry, as well as damage to some of the transverse processes. However, we do not consider that these plastic deformations could imply a great modification on the RoMs, so no retrodeformation was applied in these 3D models. Nevertheless, possible impacts of these plastic deformations on the motion have been detailed when analysing the RoMs (see below).

Díez Díaz *et al.* [3] suggested that the obtained ONP reflects the intervertebral cartilage thickness between the caudal vertebrae of the MB.R.2921 caudal series, which is roughly 10% of the centrum length (as also suggested for the neck of *Diplodocus* and *Apatosaurus* [11,13]). This information is highly important for assessing the RoMs more accurately (e.g. [51]). However, it is important to note that the intervertebral space can vary throughout the series, which could have an impact on the mobility of the different sections of the axial skeleton (see [7,52–54] and references herein). To keep the reconstructions and results easy to compare, we will stick to the previous ONP for this caudal series and refer the reader to the work of Díez Díaz *et al.* [3], where a more detailed discussion on the intervertebral space is included.

### 4.3. Range of motion analysis

**Figure 6.**
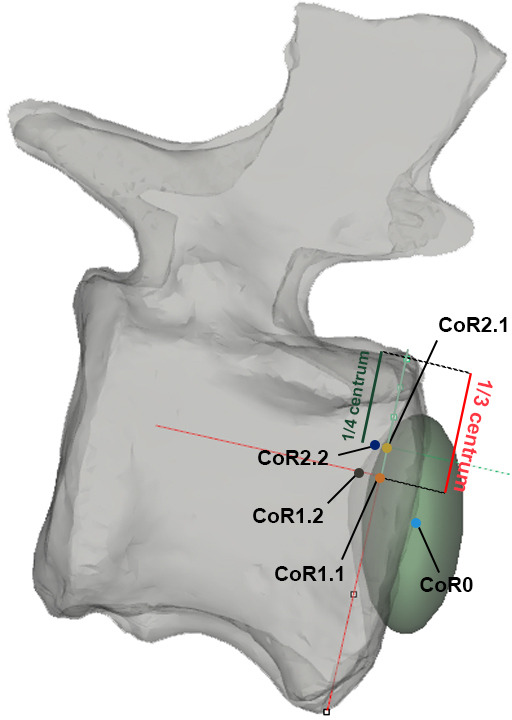
Graphical explanation of how the different CoRs have been calculated.The specimen is depicted in right lateral view, semi-transparent for a better visualization of the placement of the CoRs. For more detailed explanations, see the text. Note how CoR1.1 and CoR2.1 are located external to the centrum (in the IVD) in this specimen (MB.R.2921.10) because of its high posterior degree of concavity (2.9, see electronic supplementary material, table S2).

RoM analyses were also performed in Rhinoceros 5.0. Four copies of the ONP caudal series were created, each one for individually assessing the maximum dorsiflexion (Max-DF), maximum ventroflexion (Max-VF), maximum lateral flexion (Max-LF) and maximum torsion (Max-T). ‘Combined analysis’ *sensu* Krings *et al.* [21] and ‘cumulative RoM’ *sensu* Müller *et al.* [8] or the combination of all six degrees of motion (rotation and translation) [95–97] have not been carried out in this work. Nonetheless, we acknowledge that such combinations are necessary to capture the full mobility of the tail [95] (see §10). An ellipsoid solid element was individually created between articular surfaces as an articular joint (figure 3; a more thorough discussion on these joints is included in §3). Five different CoRs, one of them located in the geometric centroid of the ellipsoid, have been reconstructed to assess if the ranges change dramatically depending on the chosen rotation point (figures 3 and 6; see §5 for more information). Vertebral motions were achieved with the *rotation* command. Maximum motions were forced until an osteological limit appeared (i.e. complete disarticulation between post- and pre-zygapophyses of adjacent vertebrae, or points where bones in a joint would collide with each other *sensu* Wintrich *et al.* [17]; see also [14,15,19,20,50,52,57,60,98,99]. Indeed, zygapophyses (among other anatomical features [83,100]) are an important factor to stabilize the joints (see also [54]) and are significant osteological features to assess RoMs. The long axis of the first caudal vertebra and the most dorsal, ventral and lateral parts of the last vertebra of the series serve as proxies for measuring the angles in each maximum RoM (figure 6).

**Figure 7.**
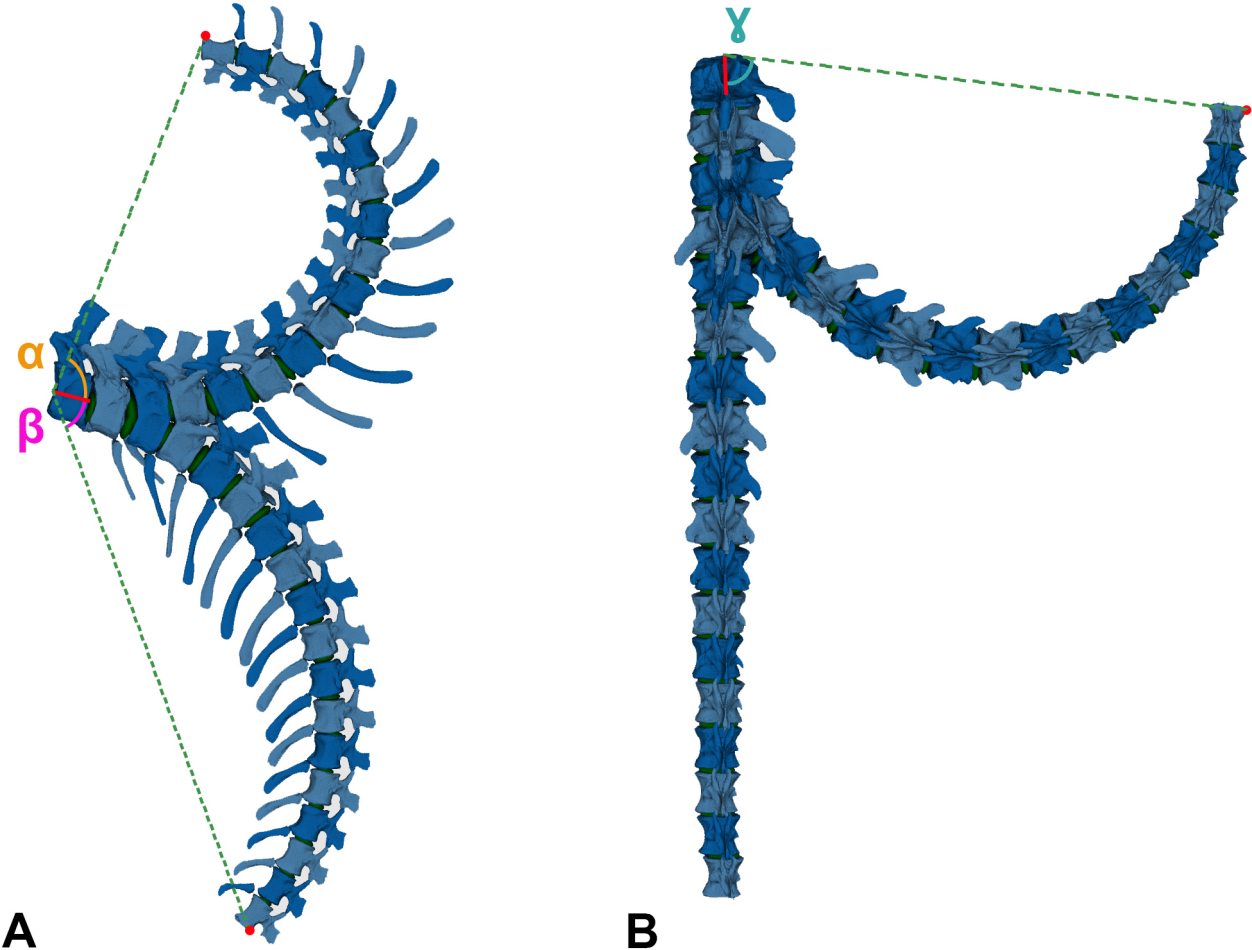
Maximum RoM angle measurements. (A) In the left lateral view, *α* describes the angle of the Max-DF, and *β* describes the angle of the Max-VF; (B) in dorsal view, *ɣ* describes the angle of the Max-LF. We measured angles made with respect to the neutral position and a reference point on the distal-most vertebra of the series. These reference points were as follows: ventral rim of the centrum (Max-DF), dorsal rim of the centrum (Max-VF) and lateral rim of the centrum (Max-LF). Not to scale.

## 5. Centres of rotation and joint articulations

The choice of CoR is a crucial factor in analysing the biomechanical properties of an axial series. Troxell [101] suggested that the position of the CoR should be located within the vertebral condyle, which will also influence results depending on the joint polarity (i.e. depending on whether the fixed element has a concave or convex posterior surface, the rotational stability will vary; see [83]). However, with amphicoelous and amphiplatyan articulations, the absence of a condyle makes identification of the CoR more challenging. Fronimos & Wilson [84] showed experimentally for *Alligator mississipiensis* that strongly convex vertebral condyles occurred in the less-flexible presacral vertebral column, while the amphiplatyan caudal vertebrae helped increase the mobility in the distal part of the tail. However, the correlation between mobility and convexity was found to be not statistically significant. Their most noteworthy hypothesis is that concavo–convex joints stabilize the axial skeleton against dislocation by shear stresses without sacrificing vertebral mobility. Salisbury & Frey [63] indicate that the dorsal osteodermal armour together with the very specialized epaxial musculature added to the stability of the axial skeleton in fossil crocodilians with amphicoelous intervertebral articulations. The same could not have been true of sauropods, which lacked a continuous carpet of osteoderms and differed from crocodilians in their axial musculature [102–104]. Dinosaurs with amphiplatyan and amphicoelous intervertebral articulations must have stabilized the axial skeleton against shear and translational stresses with zygapophyses (see [54]), powerful epaxial musculature and supraspinous and interspinous ligaments.

For MB.R.2921, the caudal series of *Giraffatitan* that we analyse in this work, we have calculated the degree of concavity of all 18 caudal vertebrae. To this end, in Rhinoceros 5.0, we drew crosshairs across the rim of each vertebral centrum and projected a line from the midpoint of the crosshair to the bone surface (figure 2; electronic supplementary material, table S2). Because the vertebrae present many small deformations and irregularities, these values are approximations. The projection point seems to coincide with the most concave area of the articulation (mean of 1.24), so all the caudal vertebrae in this series can be characterized to be amphicoelous [84]. The MB.R.2921 caudal series of *Giraffatitan* shows an interesting pattern regarding the concavity of both anterior and posterior surfaces: in the first five caudal vertebrae, the anterior concavity is deeper; in Cd6 to Cd12, it is the other way around; and in Cd13 to Cd18, the anterior and posterior concavities are similar. In contrast, most somphospondylan sauropods present more deeply concave posterior surfaces in their amphiplatyan/amphicoelous anterior and middle caudal centra (e.g. [105,106]), and in caudal vertebrae of *Haplocanthosaurus*, the anterior concavity is usually deeper than the posterior [86] (M.W. 2025, personal observation).

The choice of the location of the CoR in a planar/non-concavo–convex joint is a complex task. Fronimos & Wilson [84] suggested a ventral placement of the CoR in this kind of joints. However, several *in vivo* studies in the plane synovial joints of the lumbar section of the human axial skeleton locate the CoR of any given joint in the anterodorsal quadrant of the posterior vertebra to that joint (e.g. [59,107–109]). Cats present a similar dorsal placement of the CoR in their cervical vertebrae [110]. In mammals with amphiplatyan intervertebral articulations, such as humans, felids and lagomorphs, the instantaneous location of the CoR changes during motion. The instantaneous CoR varies in response to the instantaneous loading and the mechanical properties of the soft tissues, and the shift of CoR affects the moment arms of muscles and ligaments [110–112]. For this reason, van Bijlert *et al.* [45] tested the effects of different locations for the rotational axes (CoRs) for *T. rex* depending on whether the tail was in extension or flexion. This will only be tested to a limited extent herein.

As sensitivity analyses, we have calculated five different CoRs in each of the first 17 caudal vertebrae of *Giraffatitan*’s tail. Senteler *et al.* [59, fig. 1] projected a CoR variation grid that we have used as a starting point for our CoR calculations, so the CoR of each joint would be placed in the posterodorsal quadrant of the preceding centrum (figure 5). No experimental studies have confirmed the instantaneous location of the CoR in the posteroventral edge of the vertebrae, besides the reconstruction of forces study done by Fick [113], so this CoR placement will not be considered here. Müller *et al.* [8] found the CoR for the C7/T1 of 37 extant ruminant and camelid species and one extinct giraffid by fitting a sphere matching the curvature of the zygapophyseal facets of C7 with the sphere’s surface from the lateral and dorsal perspectives. This approach is highly interesting; however, we cannot apply it to the MB.R.2921 caudal series as the orientation of the articular facets of the postzygapophyses does not allow the creation of a sphere following their curvatures (i.e. Müller *et al*.’s [8] sample presents postzygapophyses facing ventrally and with a smooth curvature, while those of *Giraffatitan* face laterally and slightly ventrally). Following van Bijlert *et al.* [45], we have modelled the CoR0 in the midpoint of the intervertebral space, which we found by determining the geometric centroid of the ellipsoid IVD reconstruction (see above). As indicated above, we will use CoR0 as a constant CoR and do not analyse changes in the CoRs caused by redistributions of stresses. To assess if the RoMs change dramatically depending on the chosen rotation point, we have also modelled four more CoRs in the dorsoposterior quadrant of the anteriorly preceding (fixed) caudal vertebra (figures 3 and 6). CoR locations were determined as a fraction of the total dorsoventral height of the posterior articulation surface of the centrum, in sagittal view, and positioned on an anteroposterior line following the longitudinal axis of the centrum. The dorsoventral crosshair used to locate these points was defined by connecting the most dorsal and ventral points of the middle of the posterior articular surface of the centrum, effectively capturing the local height axis of that surface. Importantly, this line was not constructed as a perfect perpendicular to the longitudinal axis of the centrum. CoR1.1 and 1.2 were both placed at exactly one-third of the centrum height from the top, on the dorsal side. CoR1.1 was placed in the IVD or internally in the centrum, depending on the concavity index of the specimen, whereas CoR1.2 was projected onto the bone surface of the centrum. The anterior surface of the anterior caudal vertebrae bears a dorsal projection (lip), which becomes less noticeable from the tenth caudal vertebra onwards. This projection has its counterpart, similar to a socket where the projection fits, in the posterior surface of the preceding vertebra, affecting the mobility of the intervertebral articulation. To model the effect of this projection on the vertebral mobility, we have additionally introduced CoR2. To maintain consistency through the series, especially as this feature is difficult to discern in the posterior vertebrae, we followed the same methodology used to calculate CoR1. CoR2.1 and 2.2 were both placed at exactly one-fourth of the centrum height (from the top), on the dorsal side. CoR2.1 was also located in the IVD or internally in the centrum, depending on the concavity index of the specimen, whereas CoR2.2 was again projected onto the bone surface (figure 7).

**Figure 8.**
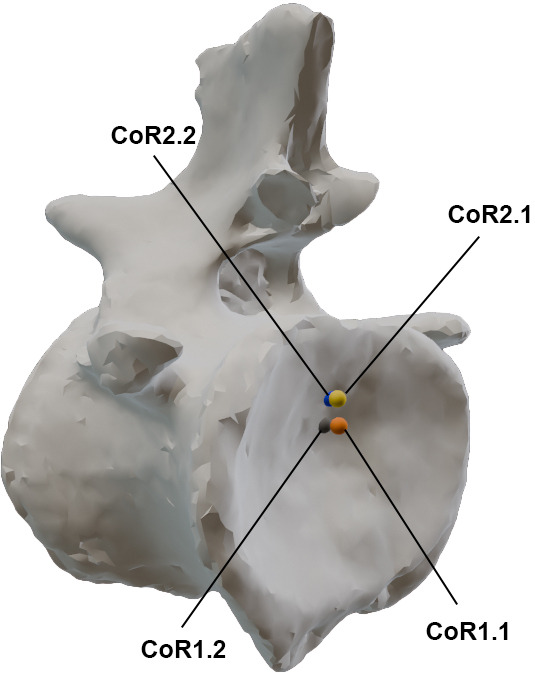
CoRs associated with the caudal centrum MB.R.2921.10. CoR1.1—orange; CoR1.2—grey; CoR2.1—yellow; CoR2.2—blue.

## 6. Biomechanical function of the haemal arches

Haemal arches (also known as chevrons) are bilaterally symmetric bony structures, positioned ventrally between two caudal vertebrae and composed of two proximal divergent rami—which can be bridged or not—and a distal blade with different degrees of development and morphologies (depending on the species and location in the caudal series; e.g. [114]). In the space between the proximal rami, several blood vessels and nerves run ventrally through the tail length, nourishing and innervating the hypaxial muscles [115,116]. The haemal arches also present osteological correlates for the attachment of hypaxial muscles [3,69].

Despite numerous studies on the anatomical features and systematic significance of the haemal arches, little is known about their biomechanical function. Only the works of Organ [117] and van Bijlert *et al.* [45] have considered them as functional units in their analyses. Haemal arches are not static elements and move together with the caudal series, but can also be mobile in themselves, as they are not fixed to the caudal vertebral centra; indeed, their articular facets and those of the centra are covered by hyaline cartilage. Due to a lack of information on their biomechanical significance in extant species, we will test in this work how the presence of haemal arches affects the ventroflexion of the tail. One model will treat the haemal arches as moving units, adapting to the motion of the tail, so they will not be considered as osteological limits. In the second model to test, the haemal arch will be considered as a fixed element moving together with the caudal vertebra, assessing their importance as osteological constraints, especially in the ventroflexion. We chose to fix the haemal arch to its attachment point on the posteriorly adjacent caudal vertebra, as morphologically the articulation to the anterior adjacent vertebra is less distinct from the intervertebral joint itself (figure 8). This *a priori* suggests an expanded gliding area for the preceding vertebra, and even maybe a larger ventral RoM. In any case, the presence of haemal arches alone indicates a degree of constraint due to the soft tissues that are attached to them.

**Figure 9.**
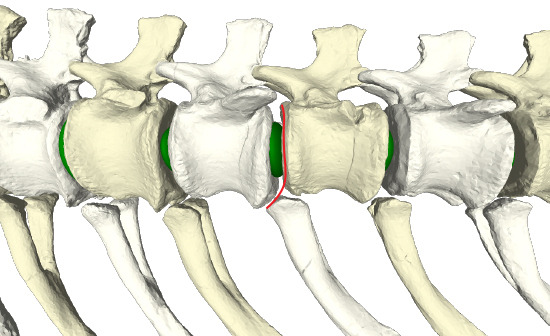
Detail on how the anterior surface of the caudal vertebra can be expanded (in red) if the anterior facet of the haemal arch is taken into account.

## 7. Ranges of motion

RoMs of the five copies of the ONP were created for each CoR (figure 5), using Rhinoceros 5.0 (electronic supplementary material, table S3). We only take into account the IVDs as cartilage tissue for the analyses, forcing the flexion and torsion until an osteological limit is reached (e.g. disarticulation and contact between zygapophyses [118,119], contact between the neural spines or centra, etc.), meaning that the synovial capsules enclosing the zygapophyses or the cartilage between the haemal arches and centra are not considered [9,50,60]. In addition, muscles, cartilage, ligaments and tendons could also have an important impact on the RoMs, so the results presented here should be interpreted as the largest possible motion range, whereas the actual RoM in the living animal was likely more restricted [9,17,19,50,57,60,63,99,118,120,121]. However, in the absence of soft-tissue data when studying extinct animals, osteological constraints form a repeatable and objective benchmark that requires no assumptions about soft tissues. Furthermore, zygapophyses limit vertebral rotation, which ensures the alignment of the vertebral centra [83], i.e. stabilize the intervertebral articulations. As a result, they are useful osteological structures for determining the osteological limits of mobility. Some previous studies assumed that sauropods arrested motion prior to disarticulation of the zygapophyses, preserving a residual overlap or zygapophyseal safety factor (approx. 25–50% zygapophyseal overlap [18]). However, some other works [15,122] have demonstrated that these values are extremely conservative when compared to the small zygapophyseal overlap needed to replicate extant vertebrate postures. In this work, we will use the complete disarticulation between zygapophyses as an osteological limit [19], where the highest possible osteological RoMs are attained.

Following Wintrich *et al*. [17], we will only consider rotational degrees of freedom, not investigating the translation between vertebrae (see also [99]). We define the rotational degrees as: *X*_*r*_ (pitch, describes a dorsoventral movement around a mediolaterally oriented axis, parallel to the anterior articulation surface of the centrum), *Y*_*r*_ (roll, describes the rotation or twist of a segment around a horizontal axis, defined by the centrum anteroposterior longitudinal axis) and *Z*_*r*_ (yaw, describes the lateral movement of a segment by rotation around a vertical axis, parallel to the anterior articulation surface of the centrum; figure 9). While *X* (pitch)_r_ is related to the dorsiflexion and ventral flexion, *Y* (roll) is related to torsion and *Z* (yaw) to lateral flexion. Abourachid *et al.* [55] and Furet *et al.* [123] found that only two (rotation) degrees of freedom could be observed in the real motion between adjacent cervical vertebrae in birds. In contrast, Kambic *et al.* [119] demonstrated that turkey cervical vertebrae are capable of rotating in all three degrees of freedom. Similarly, Jones *et al.* [57] showed that other vertebrate vertebral columns, such as those of mammals and reptiles, also exhibit mobility across all three rotational axes. Lacking this *in vivo* data for archosaurian tails, we will analyse the three rotation degrees of motion of the preserved caudal series of *Giraffatitan* here (figure 9). Two adjacent vertebrae that are connected by muscles and ligaments, together with their articulations, make up a ‘motion segment’ or ‘Junghans functional unit’, which is the smallest definable biomechanical unit in the vertebral column according to Wintrich *et al.* [17]. We work with these units in these analyses: each caudal vertebra (mobile element) was rotated with respect to the anteriorly preceding caudal vertebra (fixed element) in the series [9,50,60]. Haemal arches are considered fixed to the posterior caudal vertebra of the motion segment (see above). The angles calculated for each motion segment in each RoM and their osteological limits are indicated in the electronic supplementary material, table S3. Cd1 is kept fixed, so no data on this caudal vertebra have been gathered.

**Figure 10.**
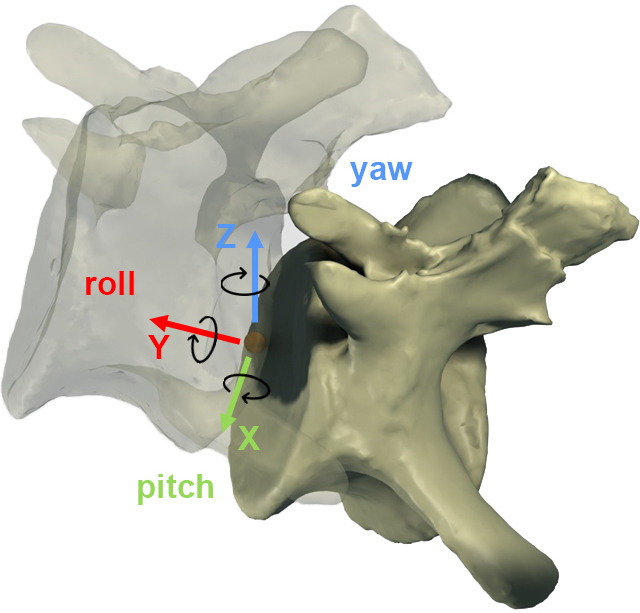
The six degrees of freedom (rotational and translational). Only rotation is analysed in this work.

### 7.1. Maximum dorsiflexion

**Figure 11.**
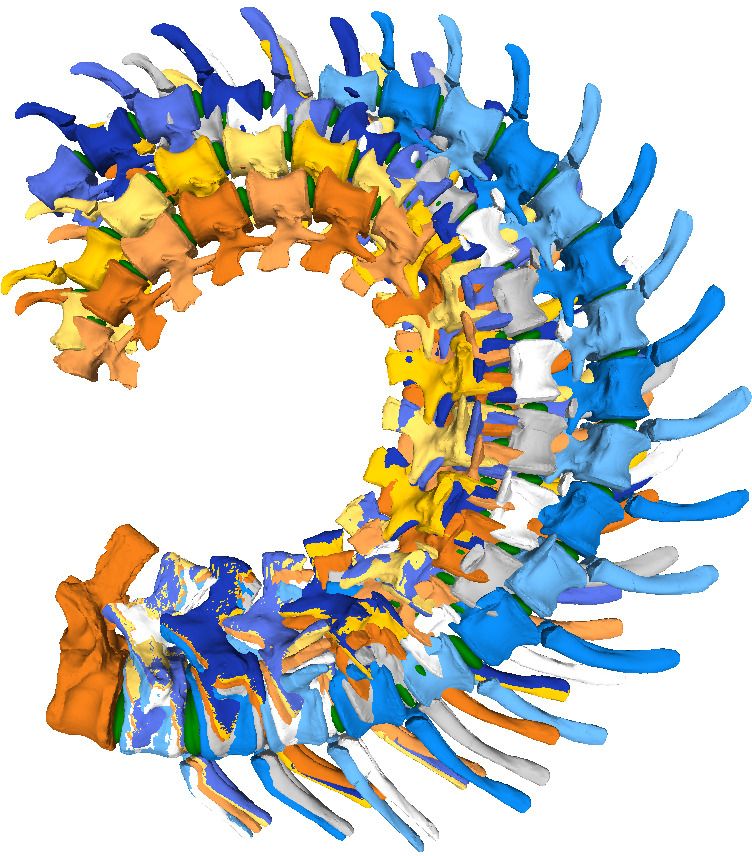
Max-DF RoMs of the MB.R.2921 caudal series of *G. brancai*. The colours of the caudal series follow the pattern of the CoRs: CoR0—light blue; CoR1.1—orange; CoR1.2—grey; CoR2.1—yellow; CoR2.2—dark blue.

**Figure 12.**
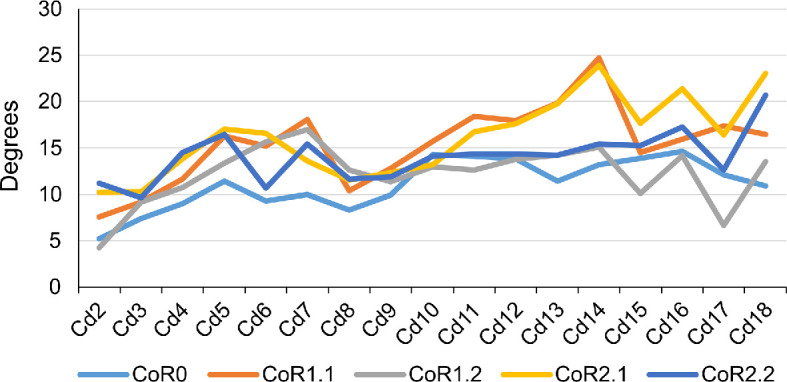
Max-DF angles of the MB.R.2921 caudal series of *G. brancai*. CoR0—light blue; CoR1.1—orange; CoR1.2—grey; CoR2.1—yellow; CoR2.2—dark blue.

The maximum total dorsiflexion was obtained with CoR2.2 (107.86°), while the lowest values were achieved with CoR0 (82.17°; figures 11 and 12; electronic supplementary material, tables S1–S3). It is noteworthy that similar degrees were obtained for all the CoRs except for CoR0. This could imply that the instantaneous CoR value does not highly influence dorsal movements when the rotation point is located in the posterodorsal quadrant of the centrum. However, when considering single centrum values, important differences can be observed. For example, CoR 1.1 and CoR2.1 RoMs present similar single values, especially from Cd9 to Cd14; this same trend also appears in the RoMs of CoR1.2 and CoR 2.2. Cd2 and Cd3 are the caudal vertebrae with the least dorsal mobility; moreover, when using CoR0, the anterior section of the tail (first nine caudal vertebrae) presents lower mobility when compared with the other vertebrae and dorsal RoMs. Maximum values are obtained for the middle to distal section of the MB.R.2921 caudal series.

The main osteological limit is the end-range articular constraint between zygapophyses in all five CoRs. Still, some isolated vertebrae and caudal sections when using CoR1.1 also show a dorsal contact between the articular surfaces of the centra. An interesting feature can be found in the first six caudal vertebrae, in which the neural spine is tall and posteriorly directed, acting like an osteological stop in some of the RoMs (mainly the contact between the neural spines of Cd2 and Cd3 and those of Cd4, Cd5 and Cd6). This limits the dorsal mobility of the base of the tail, especially when we use CoRs located in the posterodorsal quadrant of the centrum (i.e. from CoR1.1 to CoR2.2).

### 7.2. Maximum ventroflexion

**Figure 13.**
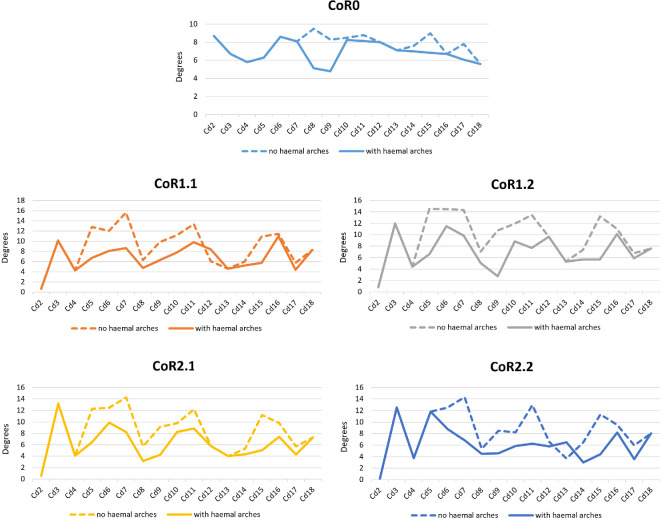
Graphics showing the differences in the ventroflexion degrees of each CoR per caudal vertebra of the MB.R.2921 caudal series of *G. brancai* when the haemal arches are fixed to their posterior caudal centra (continuous lines), or with the haemal arches considered as moving units (dashed lines).

**Figure 14.**
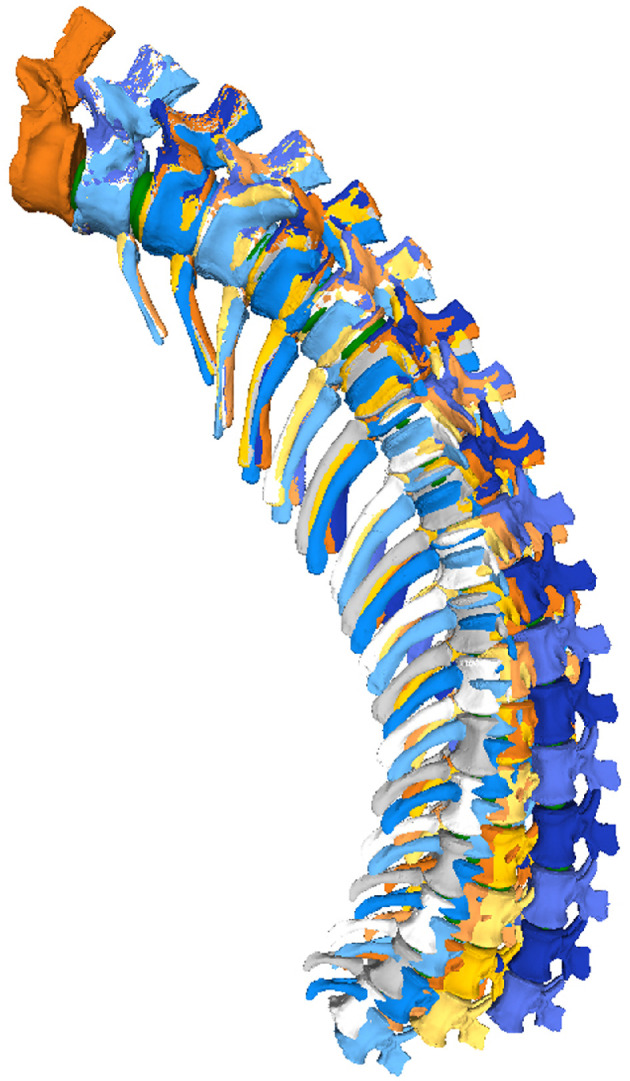
Max-VF—with fixed haemal arches – RoMs of the MB.R.2921 caudal series of *G. brancai*. The colours of the caudal series follow the pattern of the CoRs: CoR0—light blue; CoR1.1—orange; CoR1.2—grey; CoR2.1—yellow; CoR2.2—dark blue.

Due to a lack of information on their biomechanical significance in extant species, we compared the Max-VF RoMs using the haemal arches as osteological limits and as moving units (see §6; figures 12–14; electronic supplementary material, tables S3–S6). As can be seen in the electronic supplementary material, table S6 (figure 12), if each haemal arch is fixed with its posterior caudal vertebra, the angles in ventroflexion decrease and the haemal arch appears as an osteological limit in the vast majority of vertebrae (electronic supplementary material, table S3). These changes are especially dramatic when CoRs located in the dorsal quadrant of the caudal centrum are used and are less noticeable when CoR0 is chosen. This highlights the importance of considering the haemal arches when calculating the ventral RoMs, as we will obtain more restricted values and therefore more conservative results and closer to a natural state. However, it is important to remember that as these elements are not solidly fixed to the vertebral centra, they can probably adjust their position according to the restrictions they encounter during movement. This situation will not be considered in this work, as it would require further assumptions regarding ligamentous and other soft-tissue constraints. We will therefore focus on comparisons of the RoMs of the different CoRs in which the haemal arches have been fixed to the posterior caudal vertebra.

**Figure 15.**
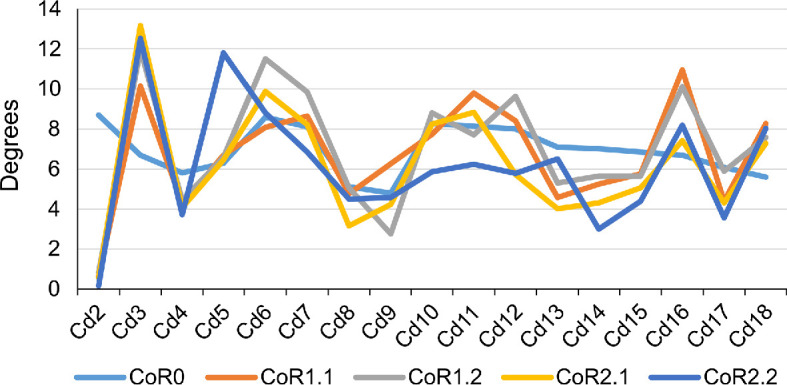
Max-VF angles of the MB.R.2921 caudal series of *G. brancai*. CoR0—light blue; CoR1.1—orange; CoR1.2—grey; CoR2.1—yellow; CoR2.2—dark blue.

The Max-VF value for MB.R.2921 was obtained for CoR1.2 (53.35°) and CoR0 (53.27°), while the lowest was achieved when using CoR2.2 (44.07°). When looking at the single centrum values, we can see that those obtained for CoR0 are more similar through the caudal series, while those calculated for the rotation points located in the posterodorsal quadrant of the centrum show a more random distribution depending on the section of the tail, but also are highly similar among the four CoRs. This indicates that when the rotation point is located in the middle of the intervertebral space, the tail shows a similar mobility pattern for all sections. In contrast, this pattern drastically changes depending on the tail section when the CoR occurs in the posterodorsal quadrant of the centrum. Nevertheless, the location of the CoR in this area of the centrum is not particularly important in terms of major variations in ventroflexion.

The main osteological limits for ventroflexion are the complete disarticulation between the zygapophyses, the contact between the ventral edge of the centra and the contact between the distal end of the haemal arches. However, the distribution of these features slightly differs among the different CoRs. For CoR0, the anterior vertebrae show as a limit to the disarticulation between the zygapophyses, while from Cd6, the haemal arches begin to play an important role as an osteological limit: the contact between their anterior surfaces and the posteroventral facets for their articulation in the anterior caudal vertebra prevents the disarticulation between the zygapophyses, limiting the ventroflexion. This tendency is even more noticeable for the other CoRs. The contact between the distal end of the haemal arches is also an important osteological limit, especially for the first and second haemal arches, which are more posteriorly oriented.

### 7.3. Maximum lateral flexion

**Figure 16.**
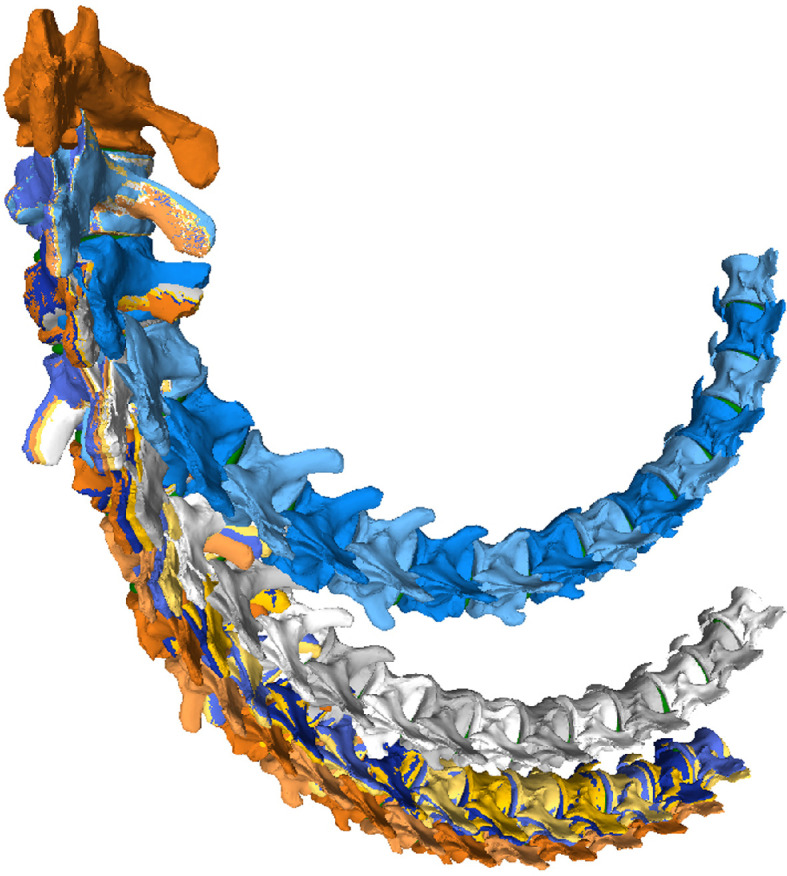
Max-LF RoMs of the MB.R.2921 caudal series of *G. brancai*. The colours of the caudal series follow the pattern of the CoRs: CoR0—light blue; CoR1.1—orange; CoR1.2—grey; CoR2.1—yellow; CoR2.2—dark blue.

**Figure 17.**
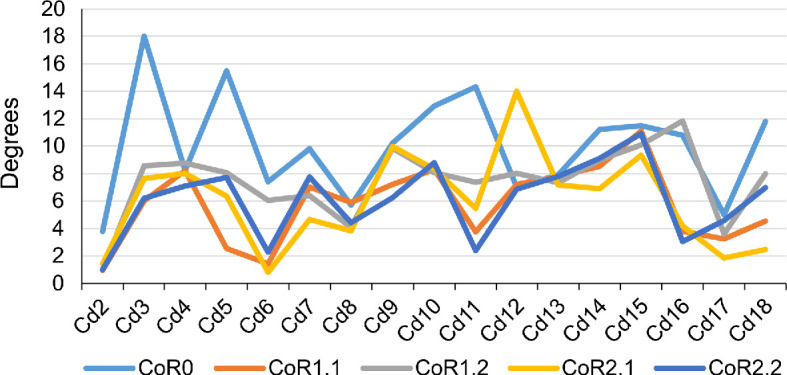
Max-LF angles of the MB.R.2921 caudal series of *G. brancai*. CoR0—light blue; CoR1.1—orange; CoR1.2—grey; CoR2.1—yellow; CoR2.2—dark blue.

The Max-LF was obtained when using CoR0 (78.80°), while the lowest was achieved for CoR1.1 (42.01°; figures 16 and 17; electronic supplementary material S3–S5). CoR2.1 and CoR2.2 also show similar lower values. No special pattern is recognizable when observing the individual caudal vertebrae values or the osteological limits, which are the lateral contact between the centra and/or the physical stop produced by the articular surface contact between zygapophyses. We can speculate that the principal osteological factor limiting the lateral movement of the tail (or increasing its lateral mobility) is again the anatomy of the zygapophyses, mainly the angle existing between them. However, the non-uniformity of these values and features in the MB.R.2921 caudal series could be related to the taphonomic distortions previously commented on, especially the posterior extension of the left postzygapophyses and the asymmetry found in several prezygapophyses, so this last hypothesis cannot be tested here confidently. We have also checked whether vertebral anatomical features might have influenced these outliers (i.e. width and height of the centre, length and angle between the prezygapophyses) and found that there were no remarkable values or links in the sample that would be indicative of them. The rim of the articulation surfaces of the centra is not completely homogeneous, so this could be another probable reason for these outliers. Here, it is also relevant to point out that CoR1.1 and 2.1 are not located on an axis completely perpendicular to *Z*_*r*_ (see §6), resulting in the lateral flexions obtained with these CoRs being slightly different.

### 7.4. Maximum torsion

The greatest summed torsion (sum of the torsion degrees of the individual joints of an axial series) is obtained with CoR1.1 (26.28°), while the lowest value occurs when CoR0 is used (11.5°; figure 17; electronic supplementary material, tables S3 and S4). The difference indicates the importance of the placement of the rotation point for torsion RoMs. Max-T angles are low, ranging from almost 0° to 1.5° when CoR0 is used and reaching 3.39° for Cd18 with CoR1.2 (the highest value among CoRs and caudal vertebrae). The preserved tail shows lower mobility when CoR0 is used. The only osteological limit is the maximum physical contact between prezygapophyseal and postzygapophyseal articular surfaces, in this case, the left ones, as each caudal vertebra is rotated clockwise. This shows again the importance of the zygapophyses for constraining the mobility of the tail, in this case, in torsion. Although the results are ‘noisy’, Cd6 to Cd14 seem to have the highest rotation values on average.

**Figure 18.**
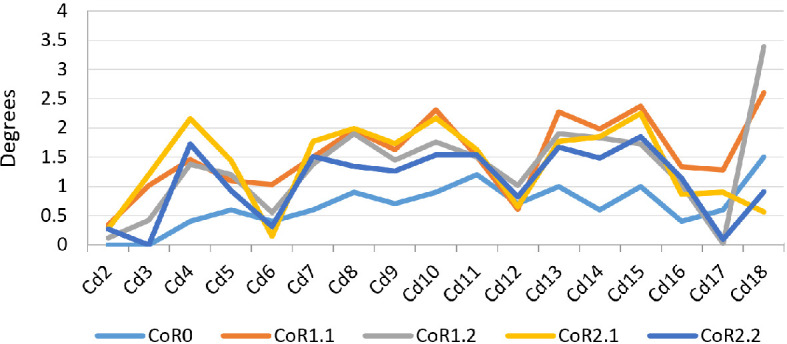
Maximum rotation angles of the MB.R.2921 caudal series of *G. brancai*. CoR0—light blue; CoR1.1—orange; CoR1.2—grey; CoR2.1—yellow; CoR2.2—dark blue.

## 8. Discussion on the centres of rotation

With these results, we can suggest that for rotational movements and dorsiflexion a more dorsal location of the CoR in the posterior surface of the caudal vertebrae of the series MB.R.2921 will produce greater RoMs, while for lateral and ventral movements, the placement of the CoR in the middle of the intervertebral space seems to produce higher values (contrasting with the results obtained by Jones *et al.* [57], in which the more dorsal location of the CoR—between the zygapophysial facets—gives higher values in lateral flexion). These results indicate that the CoR choice could dramatically influence the RoM calculations in the axial skeleton, and, therefore, subsequent biomechanical analyses and biological interpretations are necessary (see also [14,18]). The vertebrae in a macronarian caudal series can vary in their anatomical features depending on the tail section (e.g. the titanosaur *Rinconsaurus* [124]), possibly indicating that each vertebra in the series could present a different CoR location. Experimental information from extant animals is necessary to calibrate these reconstructions further.

Regarding the posterior surface concavities and the placement of the dorsal CoRs, the most noteworthy result is that ventral RoM are insensitive to CoR placement; a similar situation occurs for CoR1.1 and CoR1.2 for rotation movements. Nonetheless, it appears to be notably significant for dorsiflexion, particularly from Cd9 to Cd10 onward, and rotation RoMs for CoR2.1 and CoR2.2 (greater angles are attainable when the CoR is not required to be situated in the articular surface). No special patterns are observable for those caudal vertebrae with higher concavity values in the posterior surface (i.e. Cd6–Cd11). However, changes in the articular surfaces within the caudal series should be experimentally studied in greater detail to better understand why these changes occur and better comprehend their biomechanical functionality. Potentially interesting examples include the titanosaurs *Opisthocoelicaudia* and *Rinconsaurus* [124–126]. The anterior caudal centra of *Opisthocoelicaudia* are opisthocoelous, and its middle caudal centra are amphiplatyan–amphicoelous. The caudal series of *Rinconsaurus* shows a variation in the articular morphology of the centra, presenting pro-, opistho-, amphicoelous and biconvex centra. Indeed, this variation is considered an autapomorphy of this taxon. According to Fronimos & Wilson [84], the greater variation in caudal articular surface morphology indicates that the tail did not require the same degree of ‘safe mobility’ as the neck, possibly due to the greater number of intervertebral joints present (maximum >80 in the tail versus 18−19 in the neck) and lighter terminal load. Damage to the tail from twisting or other causes is not lethal, as the spinal cord is reduced here; in contrast, damage to the neck can result in death. Further distally in the tail, even fewer anatomical structures would be harmed.

When analysing the CoR location and maximum RoMs, it is a distinct possibility that the nerves and blood vessels or the spinal cord could be excessively compressed or stretched by the motion. Krings *et al.* [21] suggested that the diameter of the neural canal—together with the zygapophyses—could be a good criterion for assessing safe spaces for the spinal cord, as wider canals could accommodate larger rotations. These authors suggest that wide neural canals should then occur in highly mobile regions. A similar pattern is also recognizable for the height of the anterior surface of the centrum, another important osteological limit in *Giraffatitan*’s caudals series. The gentle slope shown by the change of these features in the caudal series may indicate that all caudal specimens actually exhibit *a priori* the same capacities for high mobilities for single joints and that these modifications are linked to other morphological changes (such as the orientation of the zygapophyses or the neural spine, or even pressures from soft tissues such as ligaments and muscles) that will create the changes in instantaneous CoRs or mobility. A slightly deeper valley is visible between Cd5 and Cd7 in the area of the neural canal and the length of the prezygapophyses. Nonetheless, none of the RoMs with any of the CoRs compromise the spinal cord. In general, this parameter is not usually included as a constraint when calculating RoMs, so here we highlight the importance of taking it into account in such analyses. In the ONP, the volume under the zygapophyses is sufficiently large, and in dorsiflexion, this size is maintained by the anterodorsal orientation of the prezygapophyses, preventing compression of the blood vessels and nerves in this RoM.

## 9. Discussion on the osteological limits

Using bone-to-bone contacts provides the uppermost limit for ROMs. When soft tissues are included, these ROMs will be reduced, but we are unable to better constrain these limits for the fossil taxa at present. In general, we found that the end-range articular constraint between zygapophyses is the main limit in the dorsal and lateral flexion for all CoRs. Ventral flexion tended to be restricted by the ventral contact between centra and distal parts of the haemal arches and the disarticulation between the zygapophyses (table 1). The disarticulation between the zygapophyses also seems to have more importance for the ventroflexion when the CoR is located more ventrally (i.e. for CoR0).

**Table 1.** Total percentages of each osteological constraint for the MB.R.2921 caudal series of *Giraffatitan* calculated for the four RoMs. Some limits can appear in groups of two or three.

	CoR0	CoR1.1	CoR1.2	CoR2.1	CoR2.2
centra contact	25	32	26	19	22
end-range zygapophyses	54	63	59	63	66
disarticulation zygapophyses	15	4	4	1	1
haemal arches contact	15	18	18	16	18
neural spines contact	1	0	3	3	0

## 10. Discussion on the pitch, yaw and roll

When using CoR0, the preserved tail series will achieve a slightly more symmetrical dorsoventral motion, but the pitch values are higher for the rest of the CoRs (electronic supplementary material, table S7). When looking at figure 18, an increase in the pitch can be seen from Cd5 to Cd7, while the lowest values can be found in the first four caudal vertebrae. The higher dorsal mobility of the MB.R.2921 caudal series in comparison with its ventral flexion is noteworthy, especially in the RoMs achieved with the CoRs located in the dorsal part of the centrum. As indicated above, the height of the anterior and posterior surfaces of the centrum and the anatomy of the zygapophyses are the main factors defining the pitch and the initial CoR—which could change later depending on the combined movements (‘cumulative RoMs’, see below). These findings align with previous studies on the presacral skeleton of mammals and birds, where vertebral anatomy and zygapophyseal articulation have also been shown to influence intervertebral motion (e.g. [23,127–129]).

**Figure 19.**
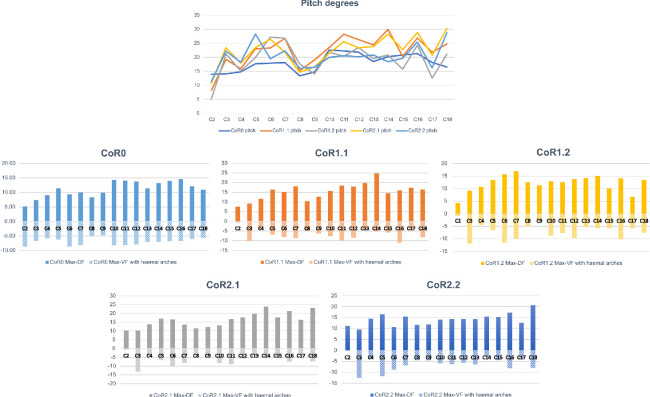
The large graphic shows the maximum pitch (dorsoventral motion) degrees that the MB.R.2921 caudal series of *G. brancai* can achieve for each CoR; CoR0—light blue; CoR1.1—orange; CoR1.2—grey; CoR2.1—yellow; CoR2.2—dark blue. The smaller graphics show the maximum pitches of each CoR; the dorsiflexion is depicted in the solid bars and the ventroflexion in the striped bars.

To simplify the analyses and to minimize asymmetries introduced by taphonomic deformations, we chose symmetrical values for lateral flexion to calculate and compare the yaw (figure 19). Larger values can be observed when the CoR0 has been chosen. All the other CoRs follow similar patterns, although similar values from Cd12 to Cd16 can be observed for almost all the CoRs.

**Figure 20.**
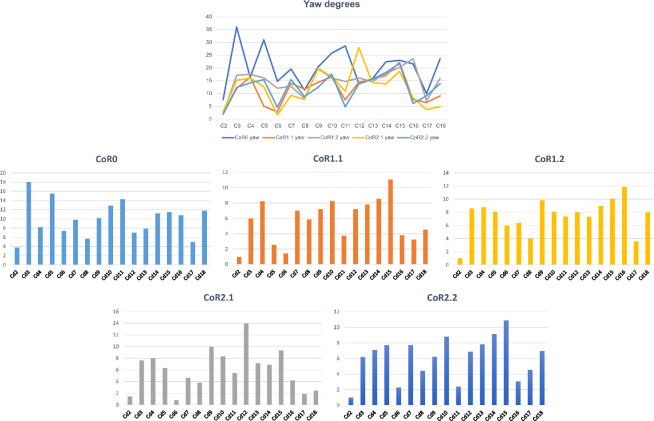
The large graphic shows the maximum yaw (lateral motion) degrees that the MB.R.2921 caudal series of *G. brancai* can achieve for each CoR. The yaw is calculated with the maximum right and left (mirrored, assuming a non-deformed caudal series) lateral motions; CoR0—light blue; CoR1.1—orange; CoR1.2—grey; CoR2.1—yellow; CoR2.2—dark blue. The smaller graphics show the maximum right lateral motion of each CoR.

As with the lateral flexion, we have also chosen symmetric values for the anticlockwise torsion (figure 20). The most anterior caudal vertebrae show low torsion values, especially Cd2 and Cd3, but from the fourth onwards, the patterns change depending on the chosen CoR. The lowest values are again found for CoR0, but the maximum roll pattern is fairly similar to that obtained for CoR1.1 and CoR1.2, showing a mobility that tends to increase towards the end of the series, with few sections with lower torsion values (e.g. Cd12, Cd16 and Cd17). When CoR2.1 and CoR2.2 are used, the low torsion degree of Cd6 and Cd12 is noteworthy, together with the lower values obtained for Cd18, especially when compared with the rest of the CoRs. These outliers are most likely due to the distortions present in the zygapophyses involved in these articulations (e.g. the postzygapophyses of Cd17 are biased towards the right side), also in conjunction with a CoR location closer to the zygapophyses.

**Figure 21.**
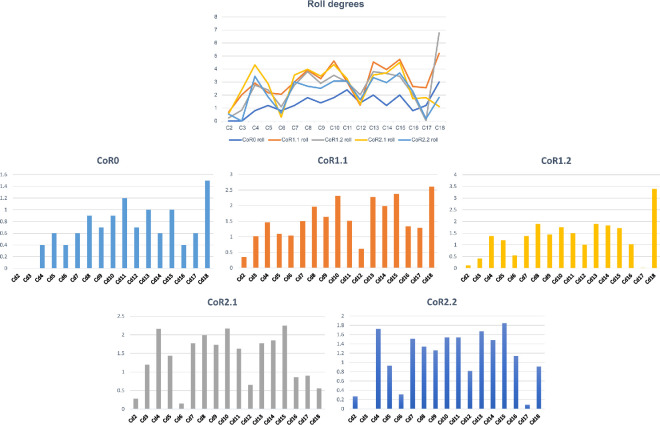
The large graphic shows the maximum roll (rotation) degrees that the MB.R.2921 caudal series of *G. brancai* can achieve for each CoR. The roll is calculated with the maximum clockwise and anticlockwise (mirrored, assuming a non-deformed caudal series) rotations; CoR0—light blue; CoR1.1—orange; CoR1.2—grey; CoR2.1—yellow; CoR2.2—dark blue. The smaller graphics show the maximum clockwise rotation of each CoR.

Finding a regionalization of the tail regarding the roll, pitch and yaw results is challenging. Generally, lower mobility of the tail can be observed in the most proximal caudal vertebrae, which makes sense when observing the general morphology of these specimens: lower average elongation index, *sensu* Chure *et al.* [130] long and posterodorsally oriented neural spines, short zygapophyses (although with broad articular surfaces), and long, posteriorly directed haemal arches. All these features have been mentioned as osteological limits for these first four vertebrae with all five CoRs. Cobley *et al.* [118] observed that once the muscles were removed from an ostrich neck, regionalization based on joint mobility was no longer achievable. This limitation is unavoidable but potentially important for our analysis of the tail of *Giraffatitan*.

We would like to highlight Merten *et al*.’s [23] statement, where they indicate that considering interactions among pitch, yaw and roll (i.e. ‘combined analysis’ *sensu* Krings *et al.* [21] and ‘cumulative RoM’ *sensu* Müller *et al.* [8], which is the sum of all plausible movements) is essential to ensure that RoM is neither underestimated nor overestimated. Moreover, combining all six degrees of freedom (rotation and translation) is necessary to ensure that true mobility is successfully captured [95–97]. Indeed, tetrapods generally use a combination of several of these degrees of freedom when moving, but as our approach is not automated and we are testing all the rotations with five different CoRs (which also change during the motion, producing different RoMs obtained with different osteological limits), we cannot confidently run these analyses and get accurate results. The results obtained (the ‘best case scenario’ as described by Stevens & Parrish [19], also without soft-tissue constraints), however, are very useful for performing comparisons with other fossil reconstructions and RoM analyses that have undergone the same methodology. In the next section, we will compare our results to six previously described titanosaurs.

The RoMs of six Late Cretaceous sauropod caudal series have been investigated to date (see electronic supplementary material, table S8; figure 21): the Spanish titanosaur *Lirainosaurus* [50], and the Brazilian taxa *Arrudatitan* [51], *Tiamat* [54], *Trigonosaurus* [52], *Adamantisaurus* and *Baurutitan *[53]*. Lirainosaurus*’ hypothetical series consists of twenty, mainly anterior and middle, caudal vertebrae, eight of them preserved as physical specimens, while the other twelve were digitally created. *Arrudatitan* caudal series presents caudal vertebrae from Cd4 to Cd9, and its RoMs were analysed in two cartilage neutral poses, with 5 and 10% of intervertebral cartilage (CNP/5% and CNP/10%) [51]. *Trigonosaurus* paratype comprises 10 caudal vertebrae, to which 10 new reconstructed specimens have been added to complete the series of the first 20 caudal vertebrae [52]. RoMs were analysed for CNP 5, 10 and 15%. *Adamantisaurus* presents a sequence of six anterior caudal vertebrae (Cd2–Cd7, RoMs analysed for CNP 5 and 10%), while *Baurutitan*’s preserved caudal series has the first 18 specimens (RoMs also analysed for CNP 5 and 10%) [53]. For *Tiamat*, only three anterior and four middle caudal vertebrae were recovered, but only the middle sequence was used for analysing the RoMs. The middle caudal sequence—probably between Cd17 and Cd27—was reconstructed modelling seven additional caudal vertebrae (see Pereira *et al.* [54] for more details on the methodology). The analysed caudal section of *Tiamat* does not allow an anatomical comparison with that of *Giraffatitan*. All these titanosaurian caudal series present concavo–convex articulations, except for the middle caudal vertebrae of *Tiamat*. Spheres were created between the caudal vertebrae for the *Lirainosaurus* analysis, in the location of the condyle, and the centre of these spheres was used as CoR (slightly above the middle of the articular surfaces) [50]. The CoR was placed over the apex of the condyle for *Arrudatitan, Trigonosaurus* and *Adamantisaurus,* which is located in the dorsal third of the vertebral body. These studies locate the CoRs slightly dorsally on the posterior surface, following the direction and development of the condyle, which corresponds to the assumptions proposed in this work for the MB.R.2921 caudal series of *Giraffatitan. Tiamat*’s middle caudal vertebrae are amphicoelous, so the authors decided to fill the space between the centra with an ellipsoid following Molnar *et al.* [88], and as we also do in this work.

**Figure 22.**
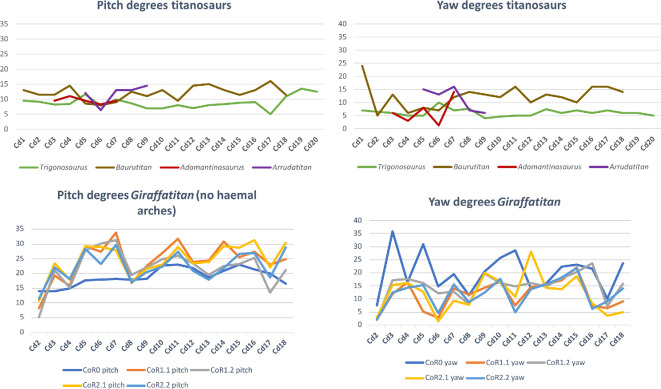
Graphics comparing the maximum pitch (dorsoventral motion) and yaw (lateral motion) degrees that the caudal series of the titanosaur taxa mentioned in the text and the MB.R.2921 one of *G. brancai* can achieve. For the *Giraffatitan* pitch, the values used are those obtained for the Max-VF with the haemal arches as moving units. Colour patterns used: *Trigonosaurus*—green; *Baurutitan*—brown; *Adamantisaurus*—red; *Arrudatitan*—purple; and for *Giraffatitan*’s CoRs: CoR; CoR0—light blue; CoR1.1—orange; CoR1.2—grey; CoR2.1—yellow; CoR2.2—dark blue.

The titanosaurs *Lirainosaurus Trigonosaurus*, *Adamantisaurus* and *Baurutitan*, as well as the MB.R.2921 caudal series of *Giraffatitan*, share a base of the tail with a low capability for dorsal motion, although this mobility slightly increases posteriorly [50, figure 3D]; [52, figure 14K]; [53, figure 8B]. In addition, more hypaxial muscles originate from and insert into the first caudal vertebrae, increasing the resistance to movement as well as the weight in this section of the tail [3]. However, the overall maximum dorsal RoMs of *Giraffatitan* are still higher than those achieved by the titanosaurian tails analysed. In *Giraffatitan*, *Lirainosaurus* and *Arrudatitan*, the disarticulation between zygapophyses was the main osteological limit, meaning that for the dorsal mobility of the tail, the morphology and development of the zygapophyses and the area of their articulation facets have great importance for both amphicoelous and procoelous centra (no specific osteological constraints are indicated for the RoMs of *Trigonosaurus, Adamantisaurus* or *Baurutitan*).

As a side note, it is interesting to mention the opisthotonic posture (i.e. dorsal retraction of the axial skeleton) that has been found in articulated skeletons of non-avian dinosaurs, fossil birds and pterosaurs (see [51] and references therein), including the sauropods *Camarasaurus*, *Tataouinea* and *Spinophorosaurus*. The preservation of these skeletons may provide further data for a better understanding of the maximum osteological dorsiflexion that can be achieved, especially in the anterior part of the caudal series, where this opisthotonia is most noticeable [51, figure 23]. However, as these authors discuss, this dorsal retraction found in the axial skeleton of these individuals is a feature still under debate—i.e. is it caused by *peri-mortem* (e.g. desiccation of the ligaments, which may cause hyperextension) or *post-mortem* (taphonomy) events?—, and therefore, the RoM values measured for these individuals should be interpreted with caution, also considering whether they are truly the maximum osteological values that can be achieved or an approximation of the rest posture of the tail in the absence of gravity.

For comparison purposes, we will use here the values obtained for the ventral RoMs using the haemal arches as moving units and not fixed to their posterior caudal vertebrae, as the reconstructed tails of *Lirainosaurus*, *Arrudatitan* and *Trigonosaurus* do not include these elements. We would like to indicate again that haemal arches are potentially important osteological limits in ventroflexion, so these values and comparisons need to be taken cautiously, as the RoMs were probably even more restricted (but see also [53]). The main osteological limits of MB.R.2921 in ventroflexion are the disarticulation between zygapophyses when CoR0 is used, as in *Lirainosaurus*. However, the main osteological limit for the more anterior caudal vertebrae of *Lirainosaurus* and *Arrudatitan*’s tail section when achieving maximum ventral RoMs is the ventral contact between centra, as in *Giraffatitan* when the haemal arches are treated as moving units. The presence of the same osteological limit may be related to the more dorsal location of the CoR, even if the intervertebral articulations have different morphologies. Here too, *Giraffatitan* is capable of achieving overall higher ventroflexion RoMs than *Lirainosaurus*, *Trigonosaurus*, *Adamantisaurus* and *Baurutitan,* especially if we compare the anterior sections of these taxa (the first 5–10 caudal vertebrae create a stiffer section [50, fig. 6C; 52, fig. 14I–K; 53, fig. 8a,b]). However, *Lirainosaurus* and *Trigonosaurus* possess a noteworthy feature in the distal anterior and middle caudal series, presenting ventrally more mobility than the other taxa. This greater ventral mobility in the midsection of the tail could have impacted their locomotor capabilities. Ventral mobility of the tail increased near the distal attachment of the *M. caudofemoralis longus*, meaning that this ventral arching could affect the moment arm of this muscle about the hip [51]. The *Giraffatitan* tail also achieves greater ventral RoMs when compared to *Arrudatitan*’s same section [51, fig. 12g,h and tables 4 and 5]. In relation to pitch, *Trigonosaurus* and *Baurutitan* present a more symmetrical dorsoventral motion in relation to those calculated for *Giraffatitan*, with more similar values between dorsiflexion and ventroflexion.

Like *Giraffatitan*, *Lirainosaurus* and *Baurutitan* show serial variation in Max-LF, while *Trigonosaurus* achieves similar values in all the caudal vertebrae. Still, *Giraffatitan* shows greater lateral RoMs than *Lirainosaurus*, *Arrudatitan* or *Trigonosaurus* CNP 5 and 10% [50, fig. 6E; 51, fig. 12c,d and tabs 4 and 5; 52, fig. 14L,M]. However, the values are highly similar when comparing the values obtained for the lateral flexion of *Giraffatitan* with CoRs 1.1, 1.2, 2.1 and 2.2 (electronic supplementary material, table S5) and those of *Trigonosaurus* CNP 15 and *Baurutitan* [52, fig. 14N; 53, fig. 8c,d]. Still, the greatest lateral flexion is achieved by *Giraffatitan* using CoR0. The slope of the zygapophyseal articulations and the angle between prezygapophyses are the main factors controlling the lateral flexion. However, in some sections of the tail of *Lirainosaurus* [50, fig. 3] and when using CoR2.2 in *Giraffatitan*, we also observe contact between the lateral edges of the centra as an osteological limit.

It is interesting to note that of all the caudal series compared in this work, *Trigonosaurus* has the most constant values throughout the series, not presenting clear sections with different capacities in terms of mobility (but also see discussion above on the regionalization of the tail without the soft tissues). However, it is important to remember that highly deviating values in the other taxa (especially the MB.R.2921 caudal series of *Giraffatitan*) could be related to anatomical asymmetries or taphonomic deformations.

Fronimos & Wilson [84] found that the vertebral articulation type (i.e. amphiplatyan, amphicoelous and concavo–convex) was not statistically significant for controlling RoM. Other anatomical features, such as the location of the CoR, as found here and as previously suggested by Stevens [18] and Vidal *et al*. [14], are more important. Our results also shed some light on the importance of the zygapophyseal articular surfaces and the length of the prezygapophyses as the main factors restricting motion (see also the works of Pittman et al. [131], Molnar *et al.* [132] and references therein for more detailed information on the features defining the joint stiffness of the axial skeleton).

## 11. Conclusion

The anterior caudal series MB.R.2921 of the Late Jurassic sauropod *G. brancai* was used to analyse its RoMs and to determine the osteological constraints on tail mobility. When dissections or *in vivo* biomechanical experiments and analyses are not feasible, or when information on soft tissues is limited, as for extinct species, these osteological limits act as proxies to more accurately calculate RoMs. It can be assumed that these maximum osteological RoMs are reduced by the addition of soft tissue. Therefore, if the contact and disarticulation protocols are maintained consistently across RoM analyses, they can provide useful comparisons among extinct animals even when their soft tissues cannot be reliably reconstructed.

This study demonstrates the importance of the selection of the instantaneous CoR and how the maximum RoMs can be affected by this choice. However, it is important to note that in life, the instantaneous CoRs not only change because of vertebral anatomy, they also vary during movement because of the redistribution of stresses. It is then important to clearly define the CoRs chosen in the analysis in order to make more objective comparisons between taxa, because choosing a fixed reference point to use for all taxa and axial series to be analysed and compared later is not possible. For this reason, in addition to clearly defining the location of the CoRs and the reasons for choosing them, the osteological constraints should also be described in detail. Future work in this area might introduce intervertebral space variability (e.g. [51–54]), use finite element analyses and/or a machine learning approach to improve accuracy and objectivity.

We found that including haemal arches as functional units is important when studying RoMs. Haemal arches are important osteological constraints on ventroflexion, and their effect is especially significant when using the more dorsally placed CoRs, as the distance to the fulcrum increases. However, as these elements are not firmly attached to the caudal centra, they most likely have the ability to shift when the tail is moving. We would also like to point out that the fossil record of haemal arches found in articulation with the caudal series is not very complete. Even when haemal arches are preserved, they are rarely described in detail. Comparing ventroflexion between analyses that include haemal arches and those that do not should be done carefully.

The information presented here could provide a foundation for explaining the role of soft tissues in stabilizing and moving the tail (i.e. a biomechanical analysis of tail function in *Giraffatitan*). In the future, we will be able to perform more reliable and objective biomechanical analyses and thus gain a better understanding of the mechanical function of the sauropod tail and its relationship to the behaviour of the animal. The sauropod tail needs to provide a stable basis for the action of *M. caudofemoralis*, the main flexor of the hip joint and hindlimb retractor [133]. Beyond that, the sauropod tail could very well have had behavioural roles, such as for defence and/or as a tactile or sensory device [134], or for communicating within the herd. As Schwaner *et al.* [2] mention, the tail extracts sensory information from its surroundings, sorts and filters it to determine the environmental relevance and then modifies its movements accordingly. During locomotion, the behaviour is quite complex because all of these steps need to be achieved while the animal is moving, thus the animal is constantly adjusting its motor output to changing visual, olfactory, auditory and mechanosensory feedback.

In conclusion, tails are more than just appendages that help sauropods move their limbs and balance their bodies; in fact, they are complex modular structures with an array of biomechanical and behavioural functions, which likely played a significant role in the development and evolutionary success of this group of animals.

## Data Availability

The 3D RoMs reconstructions simulated for each CoR are stored at MorphoSource and can be accessed following this link: https://www.morphosource.org/projects/000640950. The tables and Rhinoceros 5.0 file with the original 3D reconstruction of the ONP and RoMs simulations have been uploaded as part of the supplementary material, together with a Word file with the captions. Supplementary material is available online [135].
